# The impact of population-wide rapid antigen testing on SARS-CoV-2 prevalence in Slovakia

**DOI:** 10.1126/science.abf9648

**Published:** 2021-03-23

**Authors:** Martin Pavelka, Kevin Van-Zandvoort, Sam Abbott, Katharine Sherratt, Marek Majdan, CMMID COVID-19 working group, Intitt Zdravotnch Analz, Pavol Jaruka, Marek Kraj, Stefan Flasche, Sebastian Funk

**Affiliations:** 1 Slovak Ministry of Health, Bratislava, Slovakia.; 2 Intitt Zdravotnch Analz (Institute of Health Analyses), Bratislava, Slovakia.; 3 Department of Public Health and Policy, London School of Hygiene and Tropical Medicine, London, UK.; 4 Department for Infectious Disease Epidemiology, London School of Hygiene and Tropical Medicine, London, UK.; 5 Centre for Mathematical Modelling of Infectious Diseases, London School of Hygiene and Tropical Medicine, London, UK.; 6 Institute for Global Health and Epidemiology, Faculty of Health Sciences and Social Work, Trnava University, Trnava, Slovakia.; 7 Faculty of Medicine, Pavol Jozef afrik University, Koice, Slovakia.

## Abstract

Slovakia conducted multiple rounds of population-wide rapid antigen testing for severe acute respiratory syndrome coronavirus 2 (SARS-CoV-2) in late 2020, combined with a period of additional contact restrictions. Observed prevalence decreased by 58% (95% confidence interval: 57 to 58%) within 1 week in the 45 counties that were subject to two rounds of mass testing, an estimate that remained robust when adjusting for multiple potential confounders. Adjusting for epidemic growth of 4.4% (1.1 to 6.9%) per day preceding the mass testing campaign, the estimated decrease in prevalence compared with a scenario of unmitigated growth was 70% (67 to 73%). Modeling indicated that this decrease could not be explained solely by infection control measures but required the addition of the isolation and quarantine of household members of those testing positive.

## The Slovakian test case

Toward the end of 2020, Slovakia decided that it would test and then isolate positive severe acute respiratory syndrome coronavirus 2 (SARS-CoV-2) cases among its entire population of 5.5 million, and more than 50,000 positive cases were found during a rapid antigen testing campaign. Pavelka *et al.* analyzed the data and found that in 41 counties before and after the two rounds of testing, infection prevalence declined by about 80% (see the Perspective by Garca-Fiana and Buchan). They also used the data to test a microsimulation model for one county. Quarantine of the whole household after a positive test was essential to achieving a large reduction in prevalence. Since Autumn 2020, transmission in Slovakia has rebounded, despite other interventions, because high-intensity testing was not sustainable.

*Science*, this issue p. 635; see also p. 571

## Teaser

A nationwide testing effort in Slovakia during late 2020 reduced infection prevalence by more than 80% in 2 weeks but could not be sustained.

Nonpharmaceutical interventions have been extensively used worldwide to limit the transmission of severe acute respiratory syndrome coronavirus 2 (SARS-CoV-2) (*1*). These have included travel restrictions, mandating of face masks, closure of schools and nonessential businesses, and nationwide stay-at-home orders. All the measures were aimed at mitigating ill-health due to COVID-19 (*2*, *3*); however, they also place an unprecedented economic and social burden on the majority of uninfected people (*4*, *5*). Testing of reported symptomatic cases and tracing their contacts aims to provide a more targeted measure but, in many settings, has proven insufficient for containing transmission (*6*).

Mass testing campaigns are an alternative way to identify infectious individuals and allow the targeting of interventions without much added burden to those not infectious. However, the polymerase chain reaction (PCR) for the diagnosis of a SARS-CoV-2 infection is not suitable for mass use. Although laboratory capacities have been upscaled in record time, PCR testing remains expensive and often has turnaround times of more than 1 day, diminishing its utility (*7*). The PCR detection window also typically extends to the postinfectious period by detecting RNA fragments, hence identifying as infected those who are no longer infectious (*8*).

By contrast, rapid antigen tests are cheap and can be quickly produced in large quantities, offering results on site in 15 to 30 min without the need for a laboratory. They are less sensitive in detecting infections with low viral load that are less likely to transmit, but can detect over 70% of likely infectious cases. A recent observational study estimated the sensitivity of lateral flow devices in detecting infectious individuals to be as high as 83 to 91% (*9*). This makes mass testing a viable part of the portfolio of nonpharmaceutical interventions (*10*, *11*).

**Fig. 1:**
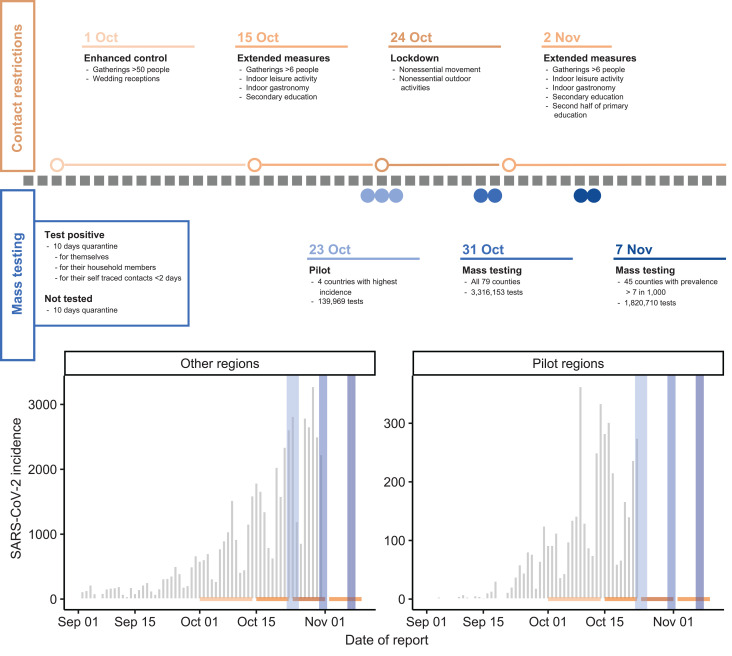
Overview of interventions and premass testing epidemiology. (**Top**) Description of timing and extent of national contact restriction in Slovakia (color intensity indicates intensity of the measures) and timing and extent of the mass testing campaigns. Open circles and lines in respective colors indicate the start and duration of the contact restrictions, and the blue solid circles indicate the days on which mass testing was conducted, although the highest turnout was usually on the first day. (**Left**) Box illustrating contact-reducing measures for those testing positive and those who chose not to be tested. (**Bottom**) SARS-CoV-2 infection incidence as reported by the Slovak Ministry of Health and collected through passive symptomtriggered PCR testing. Using the same color coding as at the top, contact interventions are indicated by horizontal lines, and mass testing campaigns are indicated by vertical lines. Data from the passive surveillance subsequent to the respective first mass testing campaign are omitted to clearly illustrate the trends in infection rates that led up to the mass testing and because mass testing is likely to have changed the sensitivity of the passive surveillance, thereby distorting the observation of infection trends that followed mass testing.

In October and November 2020, Slovakia used rapid antigen tests in a campaign that targeted the whole population to identify infectious cases at scale, rapidly reduce transmission, and thus allow easing of lockdown measures (*12*). A pilot took place between 23 and 25 October in the four most affected counties, followed by a round of national mass testing on 31 October and 1 November (round 1). High prevalence counties were again targeted with a subsequent round of testing on 7 and 8 November (round 2) (Fig. 1).

In total, 5,276,832 SD-Biosensor Standard Q rapid antigen tests were conducted by trained medical personnel during the mass testing campaigns, with 65% of the respective populations tested in the pilot, 66% in mass testing round 1 and 62% in round 2. This corresponded to 87, 83, and 84% of the age-eligible population (10 to 65 years and older adults in employment) in each round, respectively. It does not include residents who were quarantining at the time of the campaign or the 534,300 tests that were conducted on medical, military, and governmental personnel who were not included in geographical county data.

**Fig. 2:**
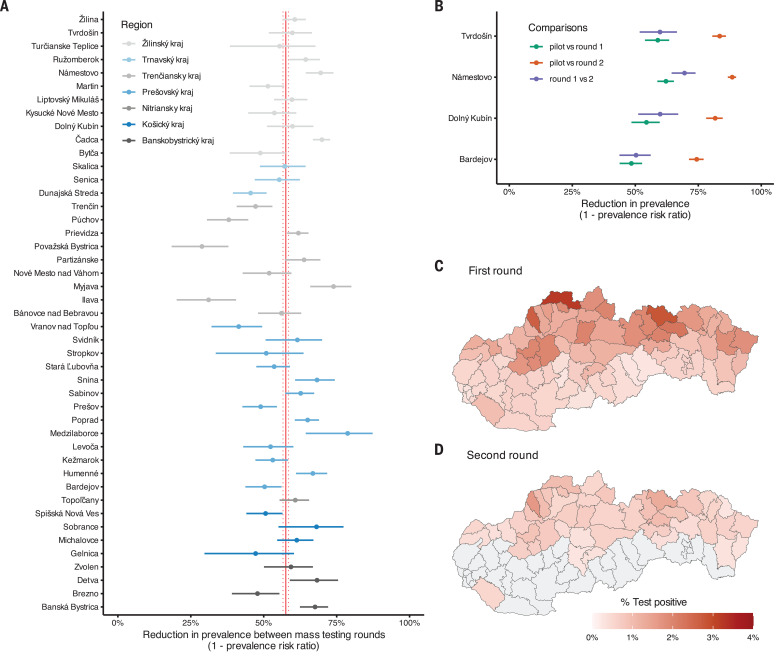
The change in test positivity between mass testing campaigns. (**A**) Change in test positivity [1 crude prevalence ratio (cPR)] observed from mass testing round 1 to round 2 in the 45 counties that were eligible for both rounds of mass testing. Counties are grouped and color coded into regions. The crude pooled estimate and its 95% confidence bounds are shown as red vertical lines. The confidence intervals were estimated using a normal approximation (Wald interval). (**B**) Change in test positivity (1 cPR) observed from the pilot mass testing round to either the first (green) or the second (orange) national round and from the first to the second mass testing round (blue) in the four counties that were included in the pilot. The confidence intervals were estimated using a normal approximation (Wald interval). (**C** and **D**) County-level test positivity in the (C) first and (D) second round of mass testing. Gray areas indicate counties that were not part of the second round because their test positivity rate was less than 7 per 1000 and hence have no estimates.

A total of 50,466 participants tested positive, indicating the presence of currently infectious SARS-CoV-2. The proportion of positive tests was 3.91% (range across counties: 3.12 to 4.84%) in the pilot, 1.01% (range: 0.13 to 3.22%) in round 1, and 0.62% (range: 0.28 to 1.65%) in round 2 (Fig. 2, C and D).

The potential for large numbers of false-positive tests has been a point of criticism for mass testing campaigns. Although multiple studies have found high specificity for the Biosensor test kit, they are not sufficiently powered to exclude specificity levels that at a population level would yield an overwhelming amount of false positives (*13*). From the low test-positive rates in some counties, we estimate with 95% certainty that the specificity of the SD Biosensor Standard Q antigen test exceeded 99.85%, and the occurrence false positives was therefore not of major concern in this study.

The counties with the highest prevalence were found in the Northern part of the country, whereas the two main Slovakian cities of Bratislava and Koice had some of the lowest observed prevalences (Fig. 1C). Reflecting this pattern, we found that high county-level prevalence was associated with a younger average population age and a lower population density (fig. S8). Given that prevalence varied at a much smaller than county scale (*14*), such associations may be clearer at the individual or community level, as observed in other countries.

In the four counties where the pilot was conducted, observed infection prevalence decreased by 56% [95% confidence interval (CI): 54 to 58%] between the pilot and round 1 of the mass testing campaign and a further 60% (95% CI: 56 to 63%) between rounds 1 and 2, totaling a decrease of 82% (95% CI: 81 to 83%) over 2 weeks. There was little heterogeneity between counties (Fig. 2B).

Among the 45 counties that were included in round 2 of the mass testing campaign, observed infection prevalence decreased by 58% (95% CI: 57 to 58%) in 1 week. Combining the pilot results with the ones from the two rounds of testing in 45 counties, each round of mass testing was estimated to have reduced observed infection prevalence by 56% (95% CI: 52 to 59%) when adjusted for attendance rates, reproduction number, and prevalence in previous rounds. The estimated reduction between rounds varied considerably by county, from 29% in county Povask Bystrica to 79% in county Medzilaborce, but although heterogeneous showed no regional pattern (Fig. 2A). Neither region, attendance rates, prevalence in round 1, nor the estimated growth rate before mass testing showed any significant impact on the observed county-specific reductions.

At the time of round 1 of the mass testing campaign, the incidence of confirmed cases reported through the syndromic surveillance system was rising in nonpilot counties, with an estimated infection growth rate of 4.4% (1.1% to 6.9%) per day. When adjusting for this growth trend, we estimated a self-adjusted prevalence ratio (saPR) of 0.30 (0.27 to 0.33). In the pilot counties, reported infection incidence showed signs of leveling in the week before the mass testing campaign, with an estimated infection growth rate of 1.3% (7.4 to 7.8%), yielding a respective saPR of 0.31 (0.26 to 0.33).

Because we used the test positivity rate of the subsequent round to estimate the impact of the previous one, we were unable to observe the impact of the last round in each county and hence the full effect of the campaign.

However, we found that the reduction achieved per round of testing was 56% (52 to 59%), indicating that the 41 counties with two rounds of testing likely reduced infection prevalence by 81% (77 to 83%) within 2 weeks and that the four counties included into the pilot testing reduced infection prevalence by 91% (89 to 93%) within 3 weeks.

The observational nature of this study made it difficult to separate the effects of the mass testing campaigns from that of the other nonpharmaceutical interventions introduced over the same period that aimed to reduce contacts and mobility, although much less than during the spring lockdown (fig. S4). Nevertheless, a greater than 50% decline in infection prevalence within 1 week (or 80% in 2 weeks) is noteworthy, particularly while primary schools and workplaces were mostly open. For comparison, a month-long lockdown in November in the UK resulted in just a 30% decrease in prevalence (*15*). This, alongside the inability in December to control the rebounding spread of SARS-CoV-2 in Slovakia through even more stringent contact restrictions, indicates that the mass testing campaigns were responsible for a large share of case reduction in the previous months.

**Fig. 3:**
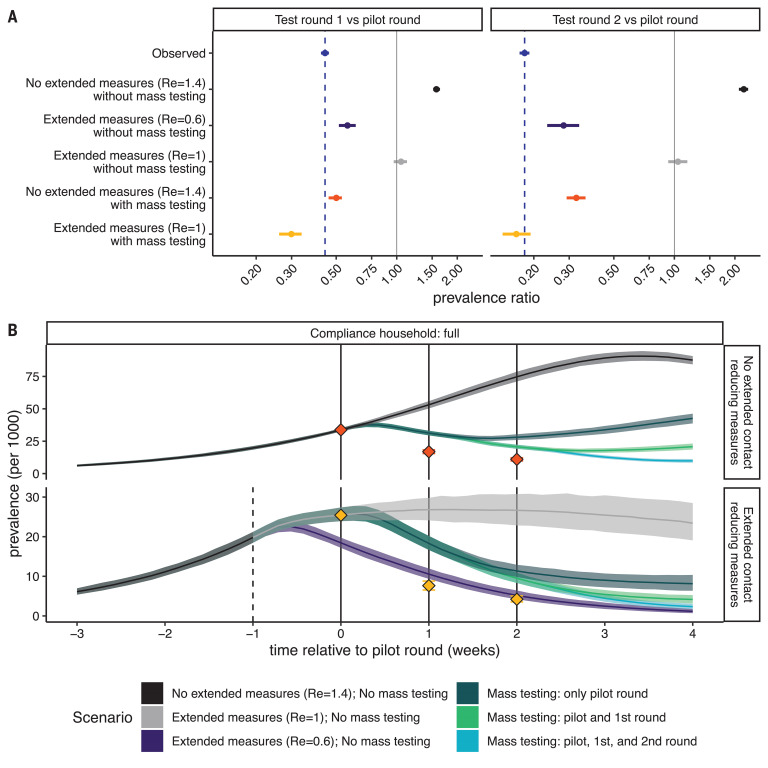
Simulated relative effectiveness of the extended contact-reducing measures and the mass testing. (**A**) The change in observed prevalence of infectious nonquarantining individuals between 10 and 65 years of age as predicted by the microsimulation model. For comparison, the observed test-positivity rate is shown in blue. The facets show changes (left) from the pilot to the first round of mass testing and (right) from the pilot to the second round of mass testing. Shown scenarios compare the effect of (top to bottom) no additional interventions that limit the growth rate of reproduction number (Re) = 1.4, the extended contact-reduction measures drastically reducing the growth rate to Re = 0.6 and no mass testing being conducted, the extended contact-reduction measures reducing the growth rate to Re = 1.0 and no mass testing being conducted, no change in growth rate but mass testing, and the extended contact reduction measures reducing the growth rate to Re = 1 and mass testing. In scenarios without mass testing, we compared prevalence of infectious individuals on the same days as testing occurred in scenarios with mass testing. CIs around the modeled values in each scenario are calculated as the 2.5 and 97.5% percentiles across 500 model iterations, with the point estimate representing the median. The CI around the observed value is its binomial CI. (**B**) Simulated infection incidence of alternative intervention strategies. Simulations are aligned by the date of the first mass test [time (*t*) = 0]. The dashed line indicates the timing of the extended contact-reducing measures, and the solid lines indicate the timing of the mass testing campaigns. Colors indicate the simulations stratified into whether no mass testing or one, two, or three testing rounds were performed and the effectiveness of the extended contact-reduction measures on the growth rate. Red and yellow diamonds indicate the prevalence of infectiousness observed among the tested nonquarantining age-eligible population, corresponding to the scenarios in (A).

To further investigate the relationship between the reduction in prevalence, mass testing, and nonpharmaceutical interventions, we used a microsimulation model for fine-scale SARS-CoV-2 transmission in a representative county included in the pilot phase of the mass testing. Among the multiple intervention scenarios tested, only the scenario that assumed a substantial impact of both the additional contact reducing measures and the mass testing campaigns was able to generate reductions in test positivity rates between testing rounds that were similar to those observed (Fig. 3). Thus, the requirement for quarantine for the whole household after a positive test was essential for the combined effect of mass testing and contact reduction measures. The model predicted a prevalence ratio between the first two testing rounds of 0.30 (0.26 to 0.34) with household quarantine and 0.78 (0.72 to 0.84) without household quarantine.

Despite a reduction of more than 50% in test positivity between mass testing campaigns, standard syndromic surveillance did not report a rapid collapse in test-positive cases corresponding to drastic reductions in prevalence. This may be explained by a variety of reasons. Foremost, the national mass testing campaigns are likely to have a major disruptive effect on routine passive syndromic surveillance. Also, the ability of PCR to detect viral RNA well beyond the infectious period will partially mask a sudden drop in infectious cases. In addition, starting mid-September incidence surveillance has been operating at capacity with long waiting lists for testing and stricter eligibility criteria, which reduced substantially in the period after mass testing and hence may have artificially reduced the observable change in these data. By contrast, data on hospital bed occupancy shows a sudden flattening from mid-November, indicating a sharp decrease in new admissions that is consistent with a sizable reduction in new infections when the mass testing campaigns occurred (fig. S6).

Executing a large-scale mass testing campaign comes with several challenges. The need to mobilize sufficient medical personnel to conduct the nasopharyngeal swabs proved to be a major obstacle. Also, the logistics of mobilizing large numbers of assisting army personnel and vast amounts of testing and personal protective equipment (PPE) material proved challenging. Some of the challenges could be overcome by using other rapid antigen tests with similarly high sensitivity but that are also licensed for use with nasal swabs (*16*, *17*). Nasal swabs can be self-administered and reduce demand on trained personnel and transmission risk in the process of sample collection and can even enable testing at home. Self-administered swabs are also less intrusive and can be better suited for children and mass testing at schools. However, these benefits must be weighed against the potential loss of sensitivity if self-administered swabs are not conducted appropriately (*18*). The details of the Slovak mass testing experience need to be studied carefully before considering potential replication elsewhere (*19*).

The combination of nationwide restrictions and mass testing with quarantining of household contacts of test positives rapidly reduced the prevalence of infectious residents in Slovakia. Although it was impossible to disentangle the precise contribution of control measures and mass testing, the latter is likely to have had a substantial effect in curbing the pandemic in Slovakia and may provide a valuable tool in future containment of SARS-CoV-2 elsewhere.

**Table 1:** Overview of county-specific test numbers and reductions for the 79 counties in Slovakia. R, median estimate of the reproduction number on 22 October, based on test-positive cases from syndromic surveillance up to 30 October and estimated by using a renewal process model on back-calculated estimates of infection incidence; %, proportion positive out of those attending mass testing.

				**Pilot**			**Round 1**			**Round 2**		
**County**	**Region**	**Population**	**R**	**Attendance**	**Positive **	**%**	**Attendance**	**Positive **	**%**	**Attendance**	**Positive**	**%**
Bnovce nad Bebravou	Treniansky kraj	36281.5	1.4				23264	457	1.96	22248	192	0.86
Bansk Bystrica	Banskobystrick kraj	110828.5	1.2				64127	687	1.07	66544	231	0.35
Bansk tiavnica	Banskobystrick kraj	16086.0	0.7				11725	33	0.28			
Bardejov	Preovsk kraj	77771.0	0.7	48320	1569	3.25	44197	740	1.67	43983	366	0.83
Bratislava I	Bratislavsk kraj	44798.0	1.2				29047	108	0.37			
Bratislava II	Bratislavsk kraj	108139.0	1.2				80958	345	0.43			
Bratislava III	Bratislavsk kraj	61418.0	1.2				49788	175	0.35			
Bratislava IV	Bratislavsk kraj	93058.0	1.2				63857	81	0.13			
Bratislava V	Bratislavsk kraj	141259.0	1.2				68139	268	0.39			
Brezno	Banskobystrick kraj	61449.5	1.4				37339	450	1.21	38515	242	0.63
Byta	ilinsk kraj	30917.0	1.6				21419	328	1.53	20931	164	0.78
adca	ilinsk kraj	90080.0	1.0				53907	1736	3.22	52304	506	0.97
Detva	Banskobystrick kraj	32051.0	1.3				19704	211	1.07	23255	79	0.34
Doln Kubn	ilinsk kraj	39456.5	1.0	29347	916	3.12	24251	345	1.42	24170	138	0.57
Dunajsk Streda	Trnavsk kraj	122358.0	1.3				87329	840	0.96	110083	577	0.52
Galanta	Trnavsk kraj	94076.0	1.3				71243	349	0.49			
Gelnica	Koick kraj	31868.0	1.3				18331	131	0.71	19087	72	0.38
Hlohovec	Trnavsk kraj	45012.5	1.4				28892	171	0.59			
Humenn	Preovsk kraj	61985.5	1.1				32962	598	1.81	32750	197	0.60
Ilava	Treniansky kraj	59187.5	1.4				37604	442	1.18	35931	291	0.81
Kemarok	Preovsk kraj	75235.0	1.4				43959	845	1.92	43252	390	0.90
Komrno	Nitriansky kraj	101711.5	1.5				61268	343	0.56			
Koice - okolie	Koick kraj	129543.5	1.2				32849	196	0.60			
Koice I	Koick kraj	67513.0	1.2				39314	295	0.75			
Koice II	Koick kraj	82287.5	1.2				11109	41	0.37			
Koice III	Koick kraj	28748.5	1.2				26992	135	0.50			
Koice IV	Koick kraj	60126.0	1.2				80426	487	0.61			
Krupina	Banskobystrick kraj	22182.0	1.4				13388	66	0.49			
Kysuck Nov Mesto	ilinsk kraj	32914.0	1.6				20605	384	1.86	20491	177	0.86
Levice	Nitriansky kraj	110824.0	1.4				70155	375	0.53			
Levoa	Preovsk kraj	33702.0	1.0				18344	373	2.03	17747	172	0.97
Liptovsk Mikul	ilinsk kraj	72260.5	1.2				47172	667	1.41	46827	267	0.57
Luenec	Banskobystrick kraj	73466.0	1.0				40655	213	0.52			
Malacky	Bratislavsk kraj	74323.0	1.3				54657	285	0.52			
Martin	ilinsk kraj	96338.0	1.5				56533	771	1.36	57513	381	0.66
Medzilaborce	Preovsk kraj	11841.5	1.1				6980	91	1.30	6142	17	0.28
Michalovce	Koick kraj	110705.0	1.0				58929	512	0.87	62790	211	0.34
Myjava	Treniansky kraj	26356.0	0.9				17753	249	1.40	18599	68	0.37
Nmestovo	ilinsk kraj	62663.5	0.9	40052	1910	4.77	37029	668	1.80	37659	207	0.55
Nitra	Nitriansky kraj	161560.0	1.3				99175	674	0.68			
Nov Mesto nad Vhom	Treniansky kraj	62553.5	1.5				40829	363	0.89	46269	198	0.43
Nov Zmky	Nitriansky kraj	139004.5	1.3				79234	478	0.60			
Partiznske	Treniansky kraj	45596.5	1.5				26492	494	1.86	27585	186	0.67
Pezinok	Bratislavsk kraj	65145.0	1.3				45801	240	0.52			
Pieany	Trnavsk kraj	62802.5	1.3				40122	183	0.46			
Poltr	Banskobystrick kraj	21471.0	2.0				12455	71	0.57			
Poprad	Preovsk kraj	104913.5	1.4				59072	1059	1.79	58098	364	0.63
Povask Bystrica	Treniansky kraj	62438.5	1.4				37822	505	1.34	36092	343	0.95
Preov	Preovsk kraj	175609.5	1.0				84781	724	0.85	108271	472	0.44
Prievidza	Treniansky kraj	133979.5	1.3				76457	1497	1.96	77170	576	0.75
Pchov	Treniansky kraj	44309.5	1.3				29455	782	2.65	28017	461	1.65
Revca	Banskobystrick kraj	39636.5	1.7				21419	58	0.27			
Rimavsk Sobota	Banskobystrick kraj	84159.0	1.7				46872	197	0.42			
Roava	Koick kraj	62208.5	1.2				34307	100	0.29			
Ruomberok	ilinsk kraj	56702.0	1.6				34000	682	2.01	33056	236	0.71
Sabinov	Preovsk kraj	60518.5	1.4				35366	804	2.27	34757	295	0.85
aa	Nitriansky kraj	51685.0	1.2				31993	199	0.62			
Senec	Bratislavsk kraj	89832.0	1.4				66052	314	0.48			
Senica	Trnavsk kraj	60446.0	1.2				40675	384	0.94	46000	194	0.42
Skalica	Trnavsk kraj	47104.5	1.2				29223	368	1.26	31200	168	0.54
Snina	Preovsk kraj	36240.5	1.3				19122	345	1.80	19396	111	0.57
Sobrance	Koick kraj	22819.0	0.9				12986	135	1.04	12966	43	0.33
Spisk Nov Ves	Koick kraj	99765.0	1.3				54279	739	1.36	53712	361	0.67
Star ubova	Preovsk kraj	53953.5	1.2				28749	805	2.80	27234	354	1.30
Stropkov	Preovsk kraj	20532.0	1.1				10494	125	1.19	10764	63	0.59
Svidnk	Preovsk kraj	32564.0	1.1				16631	220	1.32	16705	85	0.51
Topoany	Nitriansky kraj	70131.5	1.4				44627	748	1.68	50253	330	0.66
Trebiov	Koick kraj	105353.0	0.9				68503	400	0.58			
Trenn	Treniansky kraj	114523.0	1.2				73424	832	1.13	72546	434	0.60
Trnava	Trnavsk kraj	132454.5	1.2				92215	557	0.60			
Turianske Teplice	ilinsk kraj	15884.0	1.7				11287	112	0.99	12210	54	0.44
Tvrdon	ilinsk kraj	36180.0	1.3	22250	1078	4.84	18541	369	1.99	20502	164	0.80
Vek Krt	Banskobystrick kraj	43473.0	1.2				24652	76	0.31			
Vranov nad Topou	Preovsk kraj	80766.5	1.4				43552	460	1.06	45424	281	0.62
arnovica	Banskobystrick kraj	26152.5	1.4				16272	105	0.65			
iar nad Hronom	Banskobystrick kraj	46861.5	0.8				26260	108	0.41			
ilina	ilinsk kraj	158043.0	1.5				111155	1392	1.25	103898	512	0.49
Zlat Moravce	Nitriansky kraj	40572.5	0.9				26180	156	0.60			
Zvolen	Banskobystrick kraj	68758.5	1.4				39422	276	0.70	47764	136	0.28

## Supplementary Materials

science.sciencemag.org/content/372/6542/635/suppl/DC1 Materials and Methods Supplementary Text Figs. S1 to S8 Tables S1 to S3 CMMID COVID-19 Working Group Authors List Intitt Zdravotnch Analz Authors List References (*23*,*39*) MDAR Reproducibility Checklist
